# Review of strategies to reduce the contamination of the water environment by gadolinium-based contrast agents

**DOI:** 10.1186/s13244-024-01626-7

**Published:** 2024-02-27

**Authors:** Helena M. Dekker, Gerard J. Stroomberg, Aart J. Van der Molen, Mathias Prokop

**Affiliations:** 1 Department of Medical Imaging, Radboud University Medical Center, Geert Grooteplein Zuid 10, 6525 GA Nijmegen, The Netherlands; 2 RIWA-Rijn – Association of River Water Works, Groenendael 6, 3439 LV Nieuwegein, The Netherlands; 3 Department of Radiology, Leiden University Medical Center, Albinusdreef 2, 2333 ZA Leiden, The Netherlands

**Keywords:** Gadolinium-based contrast agents, Environmental fate, Water supply system, Sustainability

## Abstract

Gadolinium-based contrast agents (GBCA) are essential for diagnostic MRI examinations. GBCA are only used in small quantities on a per-patient basis; however, the acquisition of contrast-enhanced MRI examinations worldwide results in the use of many thousands of litres of GBCA per year. Data shows that these GBCA are present in sewage water, surface water, and drinking water in many regions of the world. Therefore, there is growing concern regarding the environmental impact of GBCA because of their ubiquitous presence in the aquatic environment. To address the problem of GBCA in the water system as a whole, collaboration is necessary between all stakeholders, including the producers of GBCA, medical professionals and importantly, the consumers of drinking water, i.e. the patients. This paper aims to make healthcare professionals aware of the opportunity to take the lead in making informed decisions about the use of GBCA and provides an overview of the different options for action.

In this paper, we first provide a summary on the metabolism and clinical use of GBCA, then the environmental fate and observations of GBCA, followed by measures to reduce the use of GBCA. The environmental impact of GBCA can be reduced by (1) measures focusing on the application of GBCA by means of weight-based contrast volume reduction, GBCA with higher relaxivity per mmol of Gd, contrast-enhancing sequences, and post-processing; and (2) measures that reduce the waste of GBCA, including the use of bulk packaging and collecting residues of GBCA at the point of application.

**Critical relevance statement** This review aims to make healthcare professionals aware of the environmental impact of GBCA and the opportunity for them to take the lead in making informed decisions about GBCA use and the different options to reduce its environmental burden.

**Key points**

• Gadolinium-based contrast agents are found in sources of drinking water and constitute an environmental risk.

• Radiologists have a wide spectrum of options to reduce GBCA use without compromising diagnostic quality.

• Radiology can become more sustainable by adopting such measures in clinical practice.

**Graphical Abstract:**

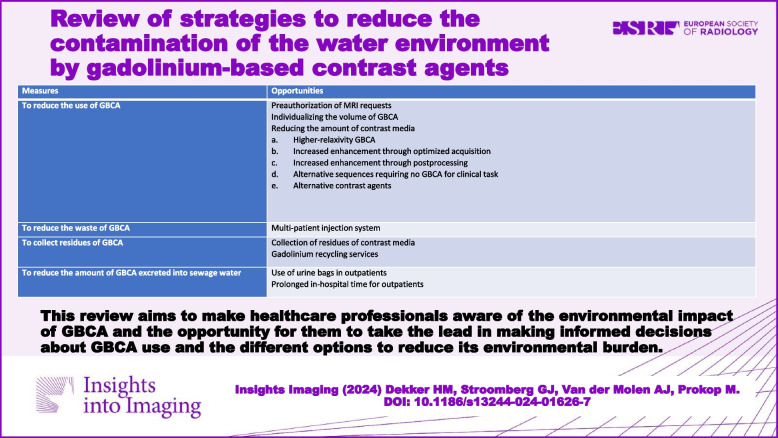

## Background

Magnetic resonance imaging (MRI) is used to help diagnose a wide range of conditions affecting the parenchymal organs, blood vessels, heart, brain, spinal cord, and bones. It can also check the health of organs such as the liver, kidneys, ovaries, breasts, and the prostate. GBCA are administrated in MRI to increase the contrast of these images. This allows radiologists to better detect inflammation, neoplasia, and functional abnormality.

Increasing use of GBCA for MRI is causing widespread contamination of fresh water and drinking water systems. GBCA can degrade, contrary to previous assumptions that they are stable throughout the water cycle. There is a need to carefully investigate the possible adverse health effects of currently marketed GBCA, and to modify the current approach to the use of GBCA in daily practice in order to minimise unknown potential health risks [1].

To tackle the problem of GBCA in the water system as a whole, it is necessary for all stakeholders, from the contrast agent manufacturer to the drinking water consumer, to work together. The first step towards active cooperation is to raise awareness among health professionals [2].

There are several things that radiologists can do to further this goal. We can be more aware of how we can optimise contrast agent use, reduce contrast agent waste, and collect contrast agent residues at the point of application. The aim of this paper is to review appropriate strategies to reduce contamination of our water supply by gadolinium-based contrast agents.

## Methods

For this narrative review, the literature was analysed using PubMed and Embase databases from January 2010 until May 2023. Multiple repetitive searches with search criteria including synonyms of “contrast agents”, “gadolinium-based”, “water”, “environment”, and “contamination” were performed for all clinically approved gadolinium-based contrast agents, with languages limited to English, Dutch, and German. Results from cross-referencing were added where appropriate.

### Gadolinium-based contrast agents

Gadolinium (Gd) is the metal located in the middle of the lanthanide series. The toxicity of Gd^3+^ in biological systems is largely due to its ability to mimic cations, particularly calcium ions (Ca^2+^), but also magnesium, zinc, and iron. These should not be overlooked, particularly because of the role of these ions as coenzymes in various biochemical processes [3]. Due to the toxic nature of Gd^3+^ ions, they are chelated with organic ligands to form GBCA that have either a linear or a macrocyclic structure. Contrast agents can therefore be classified according to the nature of the molecular structure of the ligand, either linear (i.e. open-chain molecule) or macrocyclic (i.e. cyclic ligand) and either ionic (i.e. dissociation into charged particles occurs in solution) or non-ionic [3]. The structure of GBCA determines their complex stability and in vivo stability.

#### GBCA excretion

The normal elimination of GBCA follows an open 3-compartment pharmacokinetic model. The first compartment is the plasma in which the molecules are diluted, the second compartment is the extravascular extracellular space of tissues where there is effective capillary permeability (i.e. outside the brain), and the third compartment is a storage compartment without capillary permeability. The exact location of this third compartment is not entirely clear, but GBCA are retained in the skin, liver, spleen, kidney, brain, and bone [4].

The decay of the GBCA concentration is the result of distribution of the contrast medium from plasma to the extracellular volume (distribution phase, slope α) with an average half-value of 0.2 ± 0.1 h, of the rapid excretion of GBCA from plasma to urine by renal excretion (elimination phase, slope β) with an average half-value of 1.6 ± 0.2 h, and of the slow excretion from the storage compartment to plasma and urine (residual excretion phase, slope γ). The half-value for this residual excretion phase is 5–8 × longer for linear GBCA (30–48 h) than for macrocyclic GBCA (5–6 h). The residual excretion rate is closely related to the thermodynamic stability of the specific GBCA. The relative contribution of this slow elimination is higher for linear than for macrocyclic GBCA [4].

The residence time of GBCA within the body depends also on the renal function of the patient. The half-life for the rapid renal excretion of GBCA ranges from 1.6 h for healthy individuals up to 30 h for patients with severely reduced renal function [5, 6]. In the storage compartment, the retention time of linear GBCA is long, with a risk of dechelation or transmetalation, which may lead to gadolinium deposition. Theoretically, the long residence time in the storage compartment could increase the risk of transmetalation even for macrocyclic GBCA, but to date, no clinical symptoms have been described.

#### GBCA retention and deposition

GBCA are considered to be safe and adverse effects are seldomly observed. However, in 2006, there was an association established between GBCA and nephrogenic systemic fibrosis (NSF) [7]. More recently, concerns have increased, when Gd deposits were reported in brains, bone, skin, and other tissues following GBCA administration, even in healthy patients.

In 2014, hyperintensity of the dentate nucleus (DN) and the globus pallidus (GP) relative to the pons on unenhanced T1-weighted images was attributed to repeated administrations of linear GBCA in patients with brain tumours [8], multiple sclerosis, or brain metastases [9]. No hyperintensity could be demonstrated for macrocyclic GBCA, even after large doses [10, 11]. Later studies confirmed that the use of linear extracellular GBCA led to visible changes in signal intensity (SI) ratios and measurable Gd depositions in animal and human brains [12–15], including in the anterior pituitary gland [16]. Most of the deposited Gd was found in perivascular foci in the DN and GP [12], with co-localisation to parenchymal iron [15]. The amount of deposition was not dependent on age or sex [14].

The European Medicines Agency (EMA) has therefore issued a warning and suspended the use in the EU of certain linear GBCA that are considered to have the highest risk of causing gadolinium enhancement of the brain [17].

#### Current use of gadolinium-based contrast agents

Gadopentetate dimeglumine (Magnevist), the first contrast agent developed specifically for MRI, became available for clinical use in 1988. Subsequently, several forms of GBCA from different manufacturers came onto the market.

The patents on most, if not all, of the current MRI contrast agents have expired and generics have entered the market. In the last few years, a number of generic copies of gadoterate meglumine (Dotarem®, Guerbet, Roissy CDG, France) have been registered by the regulatory authorities [18].

Gadolinium-based contrast administration is expected to continue its upward trend. This is due to the increased availability of MRI scanners and the growing number of indications for MRI examinations.

Since the introduction of GBCA in 1988, an estimated 750 million doses have been delivered, with an estimated use of 59 million doses per year in 2022. Overall, 30–45% of the MRI scans have used GBCA, largely depending on the anatomical area and specific imaging indication. There are high contributions by neuroradiology (ca. 40%) and cardiovascular radiology (ca. 20%) [19]. As a result, many thousands of litres — estimated at more than 590,000 L — of GBCA were used globally in 2022. This estimate is based on a total of approximately 59 million doses administered worldwide [19], and assuming that an average of 10 mL GBCA is used per MRI scan.

Manufacturers supply prefilled syringes, vials, and bottles in differing volumes, varying from 5 to 100 mL. According to manufacturers, there is a tendency to use more and more large packaging. In the Netherlands, we conducted a survey among the radiology departments of all hospitals, organised from the Radiological Society of The Netherlands. Of the 75 hospitals queried, 52 responded resulting in a response rate of 69%. We distinguished university hospitals, top clinical teaching hospitals, and general health organisations.

A question was raised about the ratio between the use of small packs (up to 20 mL) and larger packs (30 mL or more). More than 50% of university hospitals and top clinical teaching hospitals used bulk packs while 31% used exclusively small packs. Small packs, however, were dominant in general health organisations (58%) with bulk packs in only 23%.

### Environmental fate of gadolinium-based contrast agents

#### Gd anomaly

Gadolinium belongs to the group of rare earth elements (REEs) or lanthanides, which are found in nature as a part of rocks and minerals. Weathering of these rocks and minerals mobilises REEs which can be present as dissolved species or associated with colloids. In general, gadolinium is not enriched in natural systems compared to its neighbouring REEs because of the similar physicochemical properties of these REEs [20].

The group of REEs or lanthanides contains 15 elements, (in order of their atomic mass) La, Ce, Pr, Nd, Pm, Sm, Eu, Gd, Tb, Dy, Ho, Er, Tm, Yb, and Lu.

The REE mobilisation process is influenced by pH, salinity, flow conditions, and bedrock lithology and will determine dissolved background REE and gadolinium content, which will differ between water systems [1]. However, their natural co-occurrence is rather constant which allows for the determination of anthropogenic gadolinium by applying a correction to the total amount of measured gadolinium based on the presence of all or some REEs.

Commonly used methods to calculate natural background gadolinium concentrations are extrapolation from either the heavier REE or the lighter REE, interpolation between a heavy and a light REE (for instance Sm and Tb), or a third order polynomial fit which is the preferred option [1].

A gadolinium anomaly greater than 1.5 is defined as significant [20, 21].

Positive Gd anomalies have been found in rivers worldwide, that flow through highly populated areas, including the Dutch Rhine-Meuse Delta which shows significant positive Gd anomalies. Besides this, positive Gd anomalies have been observed ubiquitously present in lakes, estuaries and coastal waters, groundwater and tap water [22]. This is caused by GBCA.

#### Targeted analysis

A large fraction of the REEs is removed during wastewater treatment which, results in a larger Gd anomaly in the effluent which is greater than in the influent [20]. For this reason, it is preferable to detect and quantify GBCA directly, rather than the gadolinium anomaly, to assess the impact of GBCA throughout the aquatic environment. Recent method developments using hyphenated hydrophilic interaction liquid chromatography (HILIC) and inductively coupled plasma-mass spectrometry (ICP-MS) have allowed for the extraction, separation, and detection of Dotarem (Gd-DOTA), Magnevist (Gd-DTPA), Gadovist (Gd-BT-DO3A), and Multihance (Gd-BOPTA) in various stages of drinking water production ranging from surface water to finished tap water. Reported limits of detection ranged between 8 and 14 pmol/Litre [23]. A further development has been the application of fully automated Ion Chromatography separation followed by ICP-MS which allows for high-throughput screening of surface water samples. The analysis of surface water samples was suitable for five complexes (Gd-HP-DO3A, Gd-BT-DO3A, Gd-DOTA, Gd- DTPA, and Gd-BOPTA) commonly administered in the EU [24].

#### Environmental observations of GBCA

GBCA have been reported in a wide variety of environmental matrices. This is partially due to their high dosage and partially due to their relative persistence and mobility.

Table 1 shows a (non-exhaustive) list of environmental observations of GBCA or anthropogenic gadolinium in various environmental aquatic compartments.

**Table 1:** Reported GBCA contamination of the aquatic environmental compartments

Region	Locality	Type	Amount	Unit	Remarks	Reference
Wastewater						
Europe	Germany (whole)	Gd anomaly	484–1160	Kg/year	Estimated the total annual emission from hospitals	[25]
River water						
Asia	Tokyo area	Gd anomaly			5–6.6 increase over a period of 22 years	[26]
Europe	Dutch Rhine-Meuse delta	Gd anomaly	Varying concentrations		Gd mainly present in the dissolved (complexed) phase	[22]
Europe	Polish river Nida	Gd anomaly			Wastewater treatment plants as the source	[27]
Europe	German river Ems	Gd anomaly			Wastewater treatment plants as the source	[24]
Europe	Rhine River at Dutch-German border	Gadobutrol (Gd-BT-DO3A)	0.40 (avg)/0.70 (max)	ug/L	Annual (2020) averages and maximum concentrations, accounts for total Gd anomaly	[28]
		Gadoterate (Gd-DOTA)	0.43 (avg)/0.68 (max)	ug/L		
		Gadopentetate (Gd-DTPA)	Not detected			
		Gadobenate (Gd-BOPTA)	Not detected			
		Gadoteridol (Gd-HP-DO3A)	Detected, not quant			
Sea water						
North America	San Francisco Bay area and NE Pacific coast	Gd anomaly			tenfold increase between 1993 and 2013 associated with wastewater treatment plants	[29]
Pacific	Kona coast of Hawaii	Gd anomaly			Treated wastewater from Kealakehe WWTP	[30]
Europe	Atlantic Ocean	Medical Gd	5.3	Tonnes/year	Influx from European rivers	[31]
Europe	Black Sea	Medical Gd	3.0	Tonnes/year	Influx from European rivers	[31]
Europe	Mediterranean Sea	Medical Gd	2.9	Tonnes/year	Influx from European rivers	[31]
Europe	Baltic Sea	Medical Gd	1.1	Tonnes/year	Influx from European rivers	[31]
Sediment						
North America	Orlando Easterly Wetlands	Anthropogenic Gd	422–555	ng/L	From treated wastewater	[32]
Europe	Netherlands	Gd anomaly			Infiltrated surface water	[22]
Drinking water						
Europe	Germany, 6 major cities	Anthropogenic Gd			In “post-mix soda fountains” of major fast food restaurant franchises	[33]
Europe	Poland, 4 major cities	Anthropogenic Gd			Drinking water sourced from surface water influenced by wastewater treatment plants	[34]

The most important route for GBCA into the aquatic environment is through the effluent of wastewater treatment plants (WWTPs) [20, 35]. A study published in 1986, prior to the widespread use of GBCA, showed no Gd anomaly in sewage sludge from Liverpool U.K. [20, 36]. Municipal WWTP influent from large, urban areas appear to contain less anthropogenic Gd on Sundays and Mondays, possibly since most MRI exams are conducted on weekdays [20, 37].

While GBCA are relatively non-toxic, free gadolinium ions are known to cause various pathological effects [38]. Any transformation process of GBCA that creates free gadolinium would therefore greatly increase its toxic effects.

Considering the high rate of excretion, within hours of application there is little change of biotransformation by the patient. However dwelling times, in sewage systems, in wastewater treatment plants and in the aquatic environment are much longer, increasing the likelihood of transformation through biotic and a-biotic processes. Transformation may occur under aerobic or anaerobic conditions during wastewater treatment. For instance during bank infiltration as a first drinking-water purification step. Additionally, advanced oxidation processes, such as the application of ozone, are increasingly employed during wastewater treatment or drinking water purification. The impact of these processes on the stability of GBCA is yet unknown.

Another pathway for the release of free gadolinium is transmetalation, in which the complexed Gd is replaced by another metal ion. In living organisms, Zn^2+^ may be able to replace Gd^3+^ because of its relative abundance. However, some drinking water suppliers rely on Fe^3+^ as a coagulant for the removal of suspended matter by increasing flocculation. Fe(III) has a strong tendency to hydrolyse and form various hydroxo-complexes [39] lowering the ambient pH of surface water by as much as 0,5 units. This may aid the transmetalation of GBCA.

Telgmann et al. [40] described the formation of 3 unknown Gd complexes during anaerobic sewage sludge treatment. Macke et al. [24] and Okabayashi et al. [41] have detected unknown and yet unidentified Gd complexes in surface water samples from the rivers Ems (Germany) and Muko (Japan). These complexes may be the result of transformation of GBCA or transmetalation leading to the formation of other unknown Gd-complexes.

As more becomes known about the presence of GBCA in the aquatic environment, interest in their potential effects has increased. However, few studies describe the environmental effects of GBCA which can be attributed to the relative difficulty of analysing GBCA in environmental samples. Perrat et al. [42] however, describe the accumulation of GBCA in freshwater bivalves (mussels), in gills and digestive glands, downstream of a wastewater treatment at the Mosel River (France). A review by Trapasso et al. [43] highlighted the scarcity of available data regarding the effects of Gd on aquatic organisms.

## Measures to reduce GBCA

Various measures can be taken to reduce the use and the waste of GBCA, thereby reducing the amount of GBCA excreted into sewage water. Table 2 shows a list of opportunities to take measures to reduce GBCA. Below is an explanation of how to apply the measures.

**Table 2:** Measures to reduce GBCA and opportunities for implementation

Measures	Opportunities
To reduce the use of GBCA	Preauthorisation of MRI requests Individualising the volume of GBCA Reducing the amount of contrast media a. Higher-relaxivity GBCA b. Increased enhancement through optimised acquisition c. Increased enhancement through postprocessing d. Alternative sequences requiring no GBCA for clinical task e. Alternative contrast agents
To reduce the waste of GBCA	Multi-patient injection system
To collect residues of GBCA	Collection of residues of contrast media Gadolinium recycling services
To reduce the amount of GBCA excreted into sewage water	Use of urine bags in outpatients Prolonged in-hospital time for outpatients

### Measures to reduce the use of gadolinium-based contrast agents

#### Preauthorisation of MRI requests

Preauthorisation of MRI requests by a radiologist provides assessment of the correct indication for the MRI examination and the correct application of contrast medium. Preauthorisation of MRI requests and the use of GBCA leads to a significant reduction in the use of this modality, with a reduction in imaging costs and a reduction in the use of GBCA [44].

#### Individualising the volume of intravenous GBCA in contrast-enhanced MRI

More conscious use of intravenous contrast is one way of tackling the problem at source. An increasing number of institutions have introduced individualised contrast volume, based on the clinical question and personalised to body weight.

#### Reducing the amount of contrast media for contrast-enhanced MRI

The contrast dose of GBCA can be reduced if (a) the relaxivity of the Gd-compound is higher than current standards, (b) the enhancement can be increased by scanning or postprocessing techniques, (c) if the diagnostic information from contrast-enhanced sequences can be obtained without GBCA, if (d) there is a non-Gd-based alternative contrast agent available, or (e) if the (amount of) enhancement is not critical for the clinical task at hand.

##### Higher-relaxivity GBCA

The level of enhancement using GBCA depends on R1 relaxivity values and R1/R2 ratios of the GBCA [45]. Current standard compounds have a R1 relaxivity of 4.2–4.6 L/mmol s [46]. GBCA with a relatively higher in vivo relaxivity are gadobutrol (R1 in blood at 1.5 T = 5.3 L/mmol s) and gadobenate (R1 in blood at 1.5 T = 6.7 L/mmol s) [47]. The novel agent gadopiclenol has an even higher relaxivity (R1 in serum or plasma at 1.5 T = 12.8 L/mmol s) [48].

Given a standard dose of 0.1 mmol/kg with current standard compounds, correcting for differences in relaxivity theoretically results in a gadobutrol dose of ca. 0.075 mmol/kg and a gadobenate dose of 0.066 mmol/kg as the relaxivity-corrected ‘standard dose’ for these compounds. Such a relaxivity-corrected ‘standard dose’ for gadopiclenol might be even lower than the currently approved dose of 0.05 mmol/kg. However, few studies have so far performed back-to-back comparisons with this concept.

The BENEFIT study showed no significant differences between 0.05 mmol/kg gadobenate and 0.1 mmol/kg gadoterate in brain tumours [46]. For the detection of malignancy, the LEADER-75 study showed equivalent sensitivity between 0.075 mmol/kg gadobutrol and 0.1 mmol/kg gadoterate, when imaged at 1.5 T or 3 T [49]. Another study using reduced-dose gadobenate has demonstrated non-inferiority for visual lesion delineation, internal morphology, and contrast enhancement at 1.5 T and 3.0 T [50]. There is better visualisation of small or ring-enhancing metastases on half-dose delayed CE-T2w FLAIR than on half dose CE-T1w MRI [51].

Higher-relaxivity GBCA in the heart allows for using lower doses, even for cardiac MRI with late gadolinium enhancement (LGE) of myocardial ischemic scars [52–54] or for post RF ablation scars [55].

In multiple vascular territories, such as in peripheral arteries [56] or supra-aortic arteries [57], MRA (magnetic resonance angiography) with half dose gadobenate performs well. Lower doses are also suitable for time-resolved MRA [58, 59].

Reduced doses of gadobenate are non-inferior for liver MRI [60–62] and for dynamic contrast-enhancement (DCE) in prostate MRI [63]. Similar results have been achieved in breast MRI at 3 T, with either half dose gadobenate [64] or gadobutrol [65]. Half dose higher-relaxivity GBCA was sufficient for adequate 3 T imaging of bone and soft tissues in adults and children [66, 67], and for imaging synovitis in rheumatoid arthritis [68].

##### Increased enhancement through optimised acquisition

The level of enhancement of GBCA not only depends on relaxivity but also on the type of sequence and the field strength [69]. Enhancement is substantially higher at 3 T than at 1.5 T and is generally higher for T1w 3D gradient echo than for T1w 2D fast spin echo sequences. However, optimal enhancement after GBCA for a specific anatomical region and clinical indication requires careful optimisation of MRI parameters for each individual sequence. Nevertheless, this poses a potential approach to tailor the amount of contrast to field strength and minimise the use of GBCA by optimum choice of MRI sequence parameters [70].

Imaging at higher field strength allows GBCA doses to be reduced for neuroimaging. At 3.0 T a 50% dose gadopentetate has shown to give a significantly higher contrast-to-noise ratio (1.3-fold higher) than full-dose imaging at 1.5 T [71].

Nowadays, higher field strengths are the norm in abdominal MRI. Gadobenate doses may even be lowered further to a quarter dose in liver imaging in patients at risk for NSF [72].

##### Increased enhancement through postprocessing

Postprocessing using artificial intelligence techniques can create ‘virtual’ or ‘augmented’ contrast images. Augmented contrast images boost existing contrast enhancement while virtual post-contrast images use the information available on other sequences of the scan to estimate contrast-enhancement [73]. The latter only succeeds if the information is redundant, already there on other sequences.

Deep learning (DL) methods for brain MRI are still in their infancy but may reduce GBCA dose up to tenfold [74]. However, if there is no detectable enhancement in small lesions, it is very doubtful if further improvements in DL-based algorithms can solve this issue. Changes in contrast medium injection protocols may be necessary [74, 75]. Further research into the (potential) loss of diagnostic information is warranted.

Deep learning models with virtual enhancement have been used to delineate myocardial infarction areas without LGE that need the use of contrast agents [76, 77]. It can also improve contrast in low Gd-dose MRA studies in patients with congenital heart disease [78].

##### Alternative sequences requiring no GBCA for clinical task

Omitting GBCA-enhanced sequences has been studied in the follow-up of extra-axial brain masses and multiple sclerosis (MS). A meta-analysis on vestibular schwannomas showed that non-CE MRI protocols with T2w imaging are very reliable for the diagnosis and monitoring of these tumours in comparison with CE-T1w imaging [79]. Meningioma dimensions measured on pre-contrast T2w were equivalent to results on CE-T1w imaging [80].

Considering the very low incidence rate of new enhancing lesions in patients with non-progressive MS at follow-up, routine administration of GBCA in follow-up MRI is of limited value and does not change the diagnosis interval of disease progression [81]. In the French OFSEP and the MAGNIMS-CMSC-NAIMS consensus recommendations, the CE-MRI is not needed for routine follow-up but is optional for relapse or when new treatment is started [82, 83].

New MRI sequences are constantly being developed. Some provide information that renders contrast-enhanced MRI unnecessary for specific indications. Amide proton transfer (APT) imaging, for example, is a chemical exchange saturation transfer (CEST) technique offering potential clinical applications for diagnosis, characterisation, and treatment planning and monitoring in glioma patients [84]. Multiple other *specialised* non-contrast MR Angiography techniques (e.g. QISS) allow good vascular imaging without the use of GBCA and have been reviewed elsewhere [85, 86]. MR Fingerprinting has enabled GBCA-free characterisation of myocardial tissue characterisation [87].

Non-contrast MRA with steady-state free precession (SSFP) techniques allow for good quality non-contrast imaging. International guidelines state that non-CE MRA and CE-MRA are both acceptable imaging studies to measure aortic dimensions in patients with thoracic aortic disease and adults with congenital heart disease [88]. Diagnostic image quality can be achieved without the need for GBCA [89]. Non-contrast MRA, in particular balanced SSFP, works well in the analysis of renal artery stenoses [90, 91].

Time-of-flight magnetic resonance angiography (TOF-MRA) is mainly used for imaging brain arteries and carotid arteries. Phase-contrast imaging is often used in cardiac valvular flow imaging, where the data are used to quantify the shunt fraction and to assess the severity of valvular disease [92]. Arterial spin labelling (ASL) or pseudo-continuous ASL (pCASL) are mainly used in the brain for vascular and perfusion analysis, and may even allow for the depiction of a specific vascular territory by selectively labelling.

ASL perfusion imaging has several applications in the body. Taso provided an overview highlighting the ongoing challenges and solutions to enable more widespread use of this technique in clinical practice [93].

Non-contrast imaging has simplified the routine prostate MRI protocol. The resulting bi-parametric protocols (T2w and diffusion-weighted imaging) have shown good results in the diagnosis of prostate cancer [94–96]. For liver metastasis detection, non-contrast MRI with DWI was diagnostically comparable to CE-MRI protocols [97]. Non-contrast MRI is routine for 3D T2w MRCP [98], but gadoxetate T1w-MRC has added benefits for bile duct anatomy in transplantation patients [99], or to diagnose bile duct leakage following surgery or trauma [100]. More evidence is needed for non-contrast renal, pancreatic, gastro-intestinal, and adnexal MRI.

Abbreviated MRI protocols (AMRI) are now employed for several indications, either non-contrast or with GBCA. For HCC screening, non-contrast AMRI has high sensitivity and specificity, superior to ultrasound [101]. AMRI has also been used in HCC surveillance, but hepatobiliary phase AMRI has slightly better sensitivity than non-contrast AMRI because of the higher lesion-to-liver contrast [87].

Non-contrast MRI is sufficient to diagnose osteomyelitis of the appendicular skeleton [102]. However, non-contrast MRI underestimated synovitis in patients with osteoarthritis [103], and in knee synovitis CE MRI scores correlated best with inflammatory infiltrates of synovial tissue [104]. In spine MRI, contrast-enhanced sequences are better in differentiating epidural fibrosis from disc herniation [105] and for characterisation of vertebral marrow infiltrative lesions [106]. The added value of GBCA is controversial for the diagnosis of spondylitis and its complications [107].

##### Alternative contrast agents

The class of ultra-small superparamagnetic iron oxide particles (USPIOs) may be an alternative to GBCA with a high safety profile. Their iron content results in a strong T1-relaxation effect, similar to that of GBCA. One such USPIO is ferumoxytol, which was developed as a drug for anaemia in dialysis patients, but retracted from the EU markets due to severe anaphylactoid reactions. Another USPIO particle is ferumoxtran-10 (Ferrotran, SPL B.V., Nijmegen, The Netherlands), which has a high lymphotropic effect. It has been extensively evaluated for the differentiation of normal from small metastatic lymph nodes in patients with solid tumours, including prostate, bladder and breast cancer. Due to its iron content and particle size, ferumoxtran-10 has a long intravascular half-life (days) and a good safety profile and could therefore be an excellent contrast agent for MRA, especially in cases where iodine or GBCA are contraindicated, such as patients with impaired renal function and renal transplantation [108].

Another SPIO MR-contrast agent, recently available in Europe again, is ferucarbotran (Resotran, b.e.imaging GmbH, Baden-Baden, Germany) intended for liver imaging and MR-Angiography. Due to the superparamagnetic properties of the iron oxide, the contrast medium predominantly shortens the T2 relaxation time and causes a distortion of the local magnetic field, both mechanisms having a pronounced signal loss in the vicinity of the iron oxide, particularly on the T2- and T2*-weighted pictures. The T2* effect is particularly pronounced after phagocytosis of Resotran® by cells of the reticuloendothelial system (RES) during the accumulation phase. As a result, SPIO-assisted MRI distinguishes between benign and malignant lesions based on their cellular composition and function (RES cells only in normal liver tissue and benign tumours). In addition, the high T1 relaxivity of Resotran® for dynamic imaging during the vascular phase and for vascular imaging using magnetic resonance imaging Angiography (MRA) can be used [109].

From a technical point of view, already iron-containing MR-contrast media can cover the most important MR-diagnostics fields. Ferucarbotran is registered in Europe (Germany) and ferumoxtran-10 is now tested in a phase III pivotal trial for lymph node detection in prostate cancer patients, the end of the study was expected in 2023.

To the best of our knowledge, the use of USPIO in patients does not pose any known risks to the environment. This is because the USPIO contrast media are metabolised in the patient’s body after intravenous administration, and iron is a physiological element that is used in non-toxic concentrations for MR imaging. The iron from the USPIO contrast media is metabolised in the body via the normal iron metabolic cycle, and there is no known excretion of USPIOs as particles after intravenous injection.

Manganese-based contrast agents have the potential to replace GBCA, but researchers have yet to address safety adequately [110].

### Measures to reduce the waste of contrast media

#### Multi-patient injection system

The preferred method of administering the contrast agent in MRI scans is injection using a power injector. Multi-patient injection systems allow the use of vial/bottle sizes ranging from 10 to 100 mL. This allows the amount of contrast material injected to be individualised without increasing contrast material waste. The system works best by starting the day with a large bottle size (60, 65, or 100 mL) and then adjusting the bottle size at the end of the day to the expected total usage for the upcoming scan hours [111], considering the maximum usage time once the bottle stopper has been pierced. This time can vary up to 24 h, depending on the manufacturer.

A saline flush is applied. This is a secondary injection, also known as a saline chaser, following the administration of a contrast medium via a power injector.

The use of large packaging has the additional advantage of reducing overall packaging waste [112].

Manual administration is another method of administering the contrast agent. It is suitable in practices with a small number of patients for contrast-enhanced MRI (ceMRI) and do not have access to a multi-patient injection system.

### Measures to collect residues of contrast media

#### Collection of residues of contrast media

Separate collection and disposal of contrast media waste through the hospital’s waste management system prevents contrast media from entering the sewerage system [2]. At our institution, each MRI suite has a special container to collect contrast media residues. These containers are disposed of through the hospital’s dedicated waste channels and destroyed in an incinerator. Previously, these residues were simply disposed of by pouring the contrast agent down the sink.

#### Gadolinium recycling services

Gadolinium recycling is a new practice. One contrast media manufacturer [113] offers a collection and recycling service for uncontaminated contrast media leftovers. Special containers are delivered to hospital radiology departments. They are collected by the manufacturer. The gadolinium is extracted from the substance and fed it into a further industrial use, a prolongation of product lifetime.

### Measures to reduce the amount of contrast media in sewage water

#### Urine bags after contrast administration in outpatients

In The Netherlands [114] and in Germany [115] pilot studies were performed on outpatients after a contrast-enhanced CT scan. This method could also be used for outpatients after a contrast-enhanced MRI scan but no such studies of this are known to date. Disposable urine bags contain an absorbent material that holds the urine in place and can be sealed. Patients use the bags at home during the first four urination sessions after the administration of intravenous contrast media. The bags are disposed of via the household waste system*.*

#### Prolonged in-hospital time for outpatients

In Italy, the Greenwater study has started in which outpatients are asked to stay 1 h longer in hospital after a scheduled contrast-enhanced MRI examination and asked to urinate into a dedicated canister. The aim of the study is to assess the levels of GBCA in patients’ urine and to evaluate patient acceptance [116].

In The Netherlands, a study was conducted in which outpatients were asked to stay for at least 30 min longer in hospital after a scheduled ceCT examination. Patients received a urine bag for use during the first urination session. The excretion of iodinated contrast agent is roughly 20%, but varies in both terms of urine volume and the amount of contrast agent excreted [117]. Urine bags can also be applied by patients after ceMRI examinations.

## Future directions

The healthcare sector is increasingly focused on sustainability. Efforts to reduce their environmental footprint and improve sustainability are being made by both hospitals and pharmaceutical companies. The expected scarcity of raw materials and the growing demand for contrast media present a problem that must be resolved.

Manufacturers are working on reducing water consumption, energy consumption, and eco-designed packaging as well as having programmes for optimising and recovering waste at industrial plants [118].

Manufacturers have recently developed a new generation of GBCA. These are so-called high-relaxivity macrocyclic contrast agents, whereby the doses to be used can be reduced by half or even more compared to products already available on the market for a longer time.

Bracco and Guerbet jointly developed gadopiclenol. These companies will collaborate on manufacturing and research and development for indications and will commercialise gadopiclenol, independently under separate brands upon regulatory approval. FDA and EMA approvals have been obtained. The brand names are respectively Vueway (Bracco Imaging) [119] and Elucirem (Guerbet) [120]. Both contrast agents are now used in patients at a number of hospitals in the USA.

Bayer launched gadoquatrane, a tetrameric macrocyclic contrast agent [121, 122]. This contrast agent is not yet available for routine patient care, further clinical development of the drug is underway. Bayer has initiated a phase III clinical development programme called Quanti, which aims to explore the effectiveness and safety of gadoquatrane [123].

The demand for raw materials in the world continues to grow as the wealth of citizens in emerging countries increases. This, together with developments within medical imaging techniques and modern treatment options, such as in oncology, contribute to an increasing global demand for contrast media. Currently, most of the gadolinium used in the production of contrast agents is sourced from Brazil, the USA, China, India, Sri Lanka, and Australia, making the industry vulnerable to production shortages and, ultimately, the limited (and easily exploitable) natural gadolinium resources. The expected scarcity of raw materials will force more companies to adopt sustainable resource management practices. In the long term it is desirable to be able to recycle contrast media from the urine of patients, and actually producing new GBCA out of the collected used material. This helps to save raw materials and contributes to a circular process.

## Limitations

Sustainability within radiology is an evolving field and therefore limited public data is available. The measures listed to reduce the amount of gadolinium in our water environment all have their own limitations. However, taking all of these measures together may make a substantial contribution.

## Conclusions

GBCA have been found in waste, surface, and drinking water in many parts of the world. In order to tackle the problem of GBCA in the water system as a whole, it is necessary for all stakeholders, from the producer of the contrast medium to the consumer of drinking water, to work together. If everyone in the chain plays their part, environmental exposure and subsequent downstream effects can be greatly mitigated. As healthcare professionals, we must take the lead in making informed decisions about the use of GBCA.

## Data Availability

Not applicable.

## Abbreviations

AMRI: Abbreviated MRI
ASL: Arterial spin labelling
DL: Deep learning
DN: Dentate nucleus
GBCA: Gadolinium-based contrast agent/agents
Gd: Gadolinium
GP: Globus pallidus
ICP-MS: Inductively coupled plasma-mass spectrometry
LGE: Late gadolinium enhancement
MS: Multiple sclerosis
NSF: Nephrogenic systemic fibrosis
REE: Rare earth element/elements
SSFP: Steady-state free precession
USPIO: Ultra-small superparamagnetic iron oxide particle/particles
WWTP: Wastewater treatment plant/plants
